# Ovarian Reserve in Women Treated for Acute Lymphocytic Leukemia or Acute Myeloid Leukemia with Chemotherapy, but Not Stem Cell Transplantation 

**DOI:** 10.5402/2012/956190

**Published:** 2012-09-20

**Authors:** Brooke V. Rossi, Stacey Missmer, Katharine F. Correia, Martha Wadleigh, Elizabeth S. Ginsburg

**Affiliations:** ^1^Department of Obstetrics, Gynecology, and Reproductive Biology, Brigham and Women's Hospital and Harvard Medical School, Boston, MA 02115, USA; ^2^University Hospitals, Case Western Reserve University, Suite 310, 1000 Auburn Drive, Beachwood, OH 44122, USA; ^3^Channing Laboratory, Department of Medicine, Brigham and Women's Hospital and Harvard Medical School, Boston, MA 02115, USA; ^4^Department of Epidemiology, Harvard School of Public Health, Boston, MA 02115, USA; ^5^Dana-Farber Cancer Institute, Harvard Medical School, Boston, MA 02115, USA

## Abstract

*Purpose*. It is well known that chemotherapy regimens may have a negative effect on ovarian reserve, leading to amenorrhea or premature ovarian failure. There are little data regarding the effects of leukemia chemotherapy on ovarian reserve, specifically in women who received the chemotherapy as adults and are having regular menstrual periods. Our primary objective was to determine if premenopausal women with a history of chemotherapy for leukemia, without subsequent stem cell transplantation, have decreased ovarian reserve. *Materials and Methods*. We measured ovarian reserve in five women who had been treated for acute lymphocytic leukemia (ALL) or acute myeloid leukemia (AML) and compared them to age-matched control women without a history of chemotherapy. *Results*. There appeared to be a trend towards lower antimullerian hormone and antral follicle counts and higher follicle-stimulating hormone levels in the leukemia group. *Conclusion*. Our results indicate that chemotherapy for AML or ALL without stem cell transplantation may compromise ovarian reserve. Although our results should be confirmed by a larger study, oncologists, infertility specialists, and patients should be aware of the potential risks to ovarian function and should be counseled on options for fertility preservation.

## 1. Introduction

Approximately 20,000 reproductive-aged American women carry a diagnosis of leukemia [1]. For women less than 45 years old at the time of diagnosis, the 5-year survival rate is approximately 50% and 40% for women with AML and ALL. Cancer patients are not only focused on survival, but on quality of life and long-term plans, such as family building. Health care providers have recognized the importance of fertility to cancer patients and the impact of cancer treatment on fertility. The American Society of Clinical Oncology recommends that fertility preservation be discussed at the time of diagnosis [2, 3]. 

Chemotherapeutics have a range of gonadotoxic effects. In general, methotrexate, fluorouracil, vincristine, bleomycin, and dactinomycin are associated with a low or no risk of amenorrhea, which is used as a surrogate marker for infertility, while alkylating agents are more likely to cause ovarian damage and amenorrhea [2]. Alkylating agents, like cyclophosphamide, may lead to early menopause by damaging primordial follicles [4, 5]. Anthracyclines, used for induction remission and postremission chemotherapy in young adults with acute myeloid leukemia (AML) and acute lymphocytic leukemia (ALL), also likely lead to ovarian damage [6, 7]. 

Ovarian reserve refers to the number of primordial ovarian follicles in the ovaries and is related to fertility potential [8]. Infertility is more likely in women with reduced ovarian reserve [9]. Ovarian function can be monitored clinically, by the presence of regular menstrual periods. However, a menstrual cycle is not synonymous with normal fertility or ovarian reserve. In fact, women who cycle regularly may have diminished ovarian reserve [10]. Thus, biochemical tests are often employed to assess ovarian reserve. Ovarian reserve can be measured by menstrual cycle day 3 follicle stimulating hormone (FSH) and estradiol, inhibin B (INB), ovarian antral follicle count (AFC), or antimullerian hormone (AMH). 

In the current study, we examined ovarian reserve in women in first complete remission of leukemia who underwent treatment with standard chemotherapy, but who did not require stem cell transplantation. We hypothesized that premenopausal women with a history of chemotherapy for leukemia, even if stem cell transplantation is not performed and menstrual cycles are regular, have decreased ovarian reserve. 

## 2. Materials and Methods

We conducted a cross-sectional study of premenopausal women, aged 18–45, with a history of chemotherapy treatment for ALL, AML, or acute promyelocytic leukemia (APML). The Institutional Review Board of Dana Farber/Harvard Cancer Center approved this study. All participants signed informed consent and were compensated for participation. 

There was a minimum of at least 6 months since the completion of chemotherapy. Participants could be in remission or on maintenance therapy. A healthy aged-matched comparison group from the same catchment area was recruited. Women with infertility and those with medical conditions that may compromise fertility or affect ovarian reserve markers such as a history of hysterectomy or oophorectomy were excluded. Women using hormonal therapy were excluded or required to stop it for one month prior to study participation. 

Tests for ovarian reserve included serum AMH, estradiol, FSH, INB, AFC and ovarian volume. All participants were tested once on cycle days 2–4 of their menstrual cycle. The primary author performed all transvaginal ultrasounds to calculate AFC, and ovarian volumes. The AFC included all follicles in both ovaries 2–10 mm in diameter. Ovarian volume was calculated by multiplying the longest dimension of the ovary (in cm) by the two orthogonal dimensions by a factor of 0.523: ovarian volume = length × width × thickness × 0.523 [11].

We stored serum samples at −70°C. All samples were batched and concurrently analyzed. Serum estradiol was measured by the Access chemiluminescence immunoassay (Bechman Coulter, Fullerton, CA); coefficient of variation (CV) was between 21–50% for levels of estradiol 120–50 pg/mL. Serum FSH was measured by the Access chemiluminescence immunoassay (Bechman Coulter, Fullerton, CA), CV < 10%. Serum AMH was measured by enzyme-linked immunosorbent assay (ELISA) (DSL Laboratories, Fremont, CA), intra-assay CV <5% and interassay CV 6%. Serum INB was measured by ELISA (DSL Labs, Fremont, CA), intra-assay CV <5% and interassay CV 7%.

The power calculation was based on a 2-sample independent *t*-test to compare the mean AMH level between the leukemia group and the comparison group. In a previous study, the response within each subject group was normally distributed with standard deviation 0.9 ng/mL [10, 12]. If the true difference in the cancer participants and comparison means is 1.0 ng/mL, we needed to study 14 women with leukemia and 14 comparison women to be able to reject the null hypothesis that the population means of the cancer and comparison groups are equal with probability (power) 0.8. Assuming 20% loss to followup, we tried to recruit 17 leukemia patients and 17 comparison participants. However, we were only able to recruit 5 participants. Since the relapse rates are high and overall survival without a transplant is low in AML and ALL, there were a limited number of eligible patients. Our participants were aged matched (±1 year) to ensure equal age distribution in both groups. We matched the comparison participants to the leukemia participants 1 : 1. 

We compared the continuous variables (AMH, estradiol, FSH, AFC, INB, and ovarian volume) between the leukemia subjects and the comparison subjects using Wilcoxon signed-rank tests for matched pairs. In addition, we used McNemar's test based on the exact binomial distribution to look at the rate of decreased ovarian reserve markers within each group. We defined decreased ovarian reserve as menstrual cycle day 3 FSH of ≥10.0 mIU/mL, estradiol of ≥80 pg/mL, INB of <25 pg/mL, AMH <0.9 ng/mL, or AFC of <5. The type I error probability for all two-sided statistical tests was 0.05. Analyses were performed using Statistical Analysis Software (SAS) version 9.1 (SAS Institute, Inc., Cary, NC). 

## 3. Results

Five women with leukemia and five comparison women participated. At the time of study involvement, all of the participants had completed therapy; none were on maintenance therapy.  Table 1 represents their treatment history and Table 2 describes menstrual change in regards to chemotherapy. None of the participants had a history of pregnancy prior to or after chemotherapy; however, none had attempted conception. The average age of menarche was 11.6 ± 0.6 years. Sixty percent of participants used only GnRH agonist menstrual suppression during chemotherapy and the other 2 participants used combined oral contraceptive pills (COC) and a GnRH agonist. The mean duration of GnRH agonist therapy was 16 months (range 3–31 months).

Table 3 includes markers of ovarian reserve. There were no significant differences in any of the markers of ovarian reserve between the leukemia and the comparison group. However, there was a trend toward a lower AMH and AFC and higher FSH in the leukemia group. Three of the women in the leukemia group had abnormal AMH and FSH levels, compared to one in the comparison group. The 3 women with subnormal AMH levels had duration of treatment >12 months and one of these women was 35 years old at the time of treatment.

## 4. Discussion

In this age-matched study of ovarian reserve in premenopausal women who underwent chemotherapy without transplantation for acute leukemia, there were no statistically significant differences in ovarian reserve markers between the groups. However, there appeared to be a trend towards decreased ovarian reserve in leukemia survivors. Survivors may be falsely reassured that their regular menses represents normal ovarian reserve and may not seek consultation for fertility preservation until near ovarian failure. Our findings suggest that patients treated with chemotherapy for leukemia who continue to menstruate should be tested for decreased ovarian reserve after treatment. Furthermore, they should not delay consultation with an infertility specialist if they have difficulty conceiving. 

In the oncology literature, “ovarian failure” is described in a number of ways: treatment-induced amenorrhea, menopausal levels of FSH or estradiol, menopausal symptoms, or infertility. In our study, women with longer treatment durations had lower ovarian reserve. Similarly influenced by their age at treatment and the stage of cancer, approximately 50–80 percent of women that received chemotherapy for Hodgkin's lymphoma had secondary amenorrhea [13, 14]. However, 75 percent of women with a history of treatment for Hodgkin's lymphoma achieved pregnancy without assisted reproductive technologies [15]. Of note, the mean age of diagnosis was 23 years old, and those treated after the age of 30 were less likely to achieve posttreatment pregnancy. Aisner et al. found a similar pregnancy rate in their female Hodgkin's survivors, and as the length of time after therapy increased so did the probability of pregnancy [16]. Unfortunately, similar data in leukemia survivors do not currently exist outside this paper. Given the overwhelming evidence of diminished ovarian function following treatment for other hematologic malignancies, a reproductive-aged woman with a new hematologic cancer diagnosis should be aware of her potential options to retain her fertility and ovarian function [17]. Such options include oocyte, ovarian tissue, or embryo cryopreservation, which are selected based on the patient's clinical and social situation.

There are limited data on pregnancy outcomes and infertility incidence after cancer treatment. However, there are a small number of epidemiologic studies examining parenthood after a history of cancer treatment. Compared to the Norwegian general population of women, of whom 79% become parents, significantly fewer women (66%) with a history of cancer become parents. Moreover, the pregnancies of women with a history of cancer were complicated by low birth weight and premature deliveries [18]. Of note, 31% of these women had malignant melanoma (none specified as having leukemia). These findings are similar to a Finnish study that measured probability of parenthood in women diagnosed with cancer between 20–34 years old compared to their siblings [19]. The probability of parenthood in survivors compared to siblings was 38% versus 73%. Only a small proportion (3.6%) of those studied had leukemia.

There are more data on pregnancy outcomes of women diagnosed with leukemia as children than women diagnosed as adults, which reflects the improved survival rate in this age group compared to adults with acute leukemia. Among participants in the Childhood Cancer Survivor Study, the chance of live birth was significantly less (RR 0.52, 95% CI 0.36–0.76) for women treated with chemotherapy compared to their healthy siblings [20]. For women who did deliver, offspring were more likely to be of low birth weight. Signorello et al. also found that survivors were nearly twice as likely to have premature deliveries [21]. The aforementioned studies describe pregnancy outcomes and parenthood for many types of cancer, but leukemia patients are not well represented. Leukemia survivors are a large part of the Childhood Cancer Survivor Study participants, but these females were diagnosed when younger than age 22. Thus, the oncology and fertility literature lacks information on ovarian reserve after adult diagnosis and treatment of leukemia. 

Another aim of our study was to determine which ovarian reserve markers performed best in this population. The optimal ovarian reserve testing may be different for cancer survivors than for women with other infertility etiologies. In a study of Childhood Cancer Survivors, women with normal FSH levels (<10 mUI/mL) and normal menstrual cycles were found to have a lower AFC and ovarian volumes compared to age-matched controls [22]. Similarly, AMH levels were lower in women treated as children with chemotherapy for Hodgkin's lymphoma, even in women with normal FSH and INB levels. Thus, AMH appears to be an earlier and more sensitive marker of ovarian reserve in women with a history of chemotherapy [10]. Our study also showed a trend towards low AMH and AFC, with normal FSH in the leukemia group. 

Unlike FSH or estradiol, the interpretation of AMH is not menstrual cycle-day dependent [23]. During and after treatment, she may be amenorrheic from the chemotherapy or GnRH agonist use, and her cycle day unknown. Also, she often cannot postpone urgent chemotherapy for a menstrual cycle day 3 FSH evaluation. Thus, the ability to evaluate ovarian reserve regardless of menstrual status suggests that AMH is an excellent ovarian reserve marker for the cancer population. 

Current options for fertility preservation in cancer patients receiving chemotherapy include embryo cryopreservation after in vitro fertilization (IVF), oocyte cryopreservation, and ovarian suppression with gonadotropin releasing hormone (GnRH) agonists or antagonists [2]. In addition, ovarian tissue cryopreservation, currently experimental, may be considered more in the future [24]. Aside from IVF potentially being cost or time prohibitive for young women, especially those needing urgent chemotherapy for acute leukemia, IVF and embryo cryopreservation may not be desirable to single women, as they would need donor sperm. They may consider oocyte cryopreservation; however, this is still thought to be experimental, requires ovarian stimulation, and may not be feasible in the acute leukemia population because of the need to delay chemotherapy [25]. Many providers offer women GnRH agonists, as it may be covered by insurance and can be given without delaying chemotherapy. However, in 2006, the American Society of Clinical Oncology stated there was “insufficient evidence regarding the safety and effectiveness of GnRH analogs and other means of ovarian suppression on female fertility preservation [2].”

The primary limitation of this study was its size and we encourage providers to consider this when interpreting the results. Although our participants were from an academic center in a large city, we still had difficulty with recruitment. A multicenter study is required to achieve recruitment for an adequately powered study. Low numbers of eligible patients may reflect that very few women with leukemia are able to be cured with chemotherapy alone. For those who have a good prognosis, and may not need transplantation, our study suggests that there may be long-term implications regarding fertility. Due to the lack of data in the field regarding the impact of leukemia treatment on menstrual cycles and infertility, we feel that our study is a worthwhile contribution and may assist with patient counseling. We believe that women treated with chemotherapy for leukemia should be counseled that their ovarian reserve may be negatively impacted.

In conclusion, we determined that premenopausal women who have received chemotherapy as adults for leukemia may have compromised ovarian reserve, even in the setting of regular menses. Oncologists, infertility specialists, and patients should consider our results when discussing the impact of chemotherapy and fertility preservation. Further, women and providers may be able to have a more realistic conversation about the options for fertility preservation.

## Figures and Tables

**Table 1:** Chemotherapy regimens.

Subject	Diagnosis	Age at study participation	Age at diagnosis	Duration of chemotherapy (months)	Chemotherapy regimen
					Induction: prednisone, vincristine, doxorubicin, MTX, L-asparaginase
					CNS therapy: cranial radiation, vincristine, doxorubicin, 6-MP, IT MTX/ARA-C/hydrocortisone
1	ALL	39	35	14	Intensification therapy cycle 1–7: vincristine, dexamethasone, 6-MP, doxorubicin, asparaginase
					Intensification therapy cycle 8: vincristine, dexamethasone, 6-mercaptopurine, MTX
					Maintenance: vincristine, MTX, prednisone, 6-MP

2	AML	33	29	3	7 + 3 induction
					HiDAC consolidation cycle 1 and 2

					Induction: ATRA + idarubicin
3	APML	33	29	19	Consolidation cycle 1: ATRA, idarubicin
					Consolidation cycle 2: ATRA, mitoxantrone Consolidation cycle 3: ATRA, idarubucin
					Maintenance: ATRA, 6-MP, MTX × 12 months

					Induction: 7 + 4 induction with ATRA
4	APML	27	25	7	Consolidation 1 and 2: arsenic trioxide
					Consolidation cycle 3 and 4: daunorubicin and ATRA
					Cycle 4: ATRA, MTX, 6MP × 12 months

					Prophase and induction: methylprednisolone, IT ARA-C, prednisone, vincristine, doxorubicin, MTX, PEG-asparaginase, IT MTX/ARA-C/hydrocortisone, IT MTX/hydrocortisone
					Consolidation IA: vincristine, doxorubicin, IT MTX/hydrocortisone, high-dose MTX, 6-MP
					Consolidation IB: cyclophosphamide, IT MTX/hydrocortisone, ARA-C, 6-MP
5	ALL	25	21	21	Consolidation IC: HiDAC, etoposide, dexamethasone, PEG-asparaginase
					PEG-asparaginase during consolidation IC and continued through CNS phase and consolidation II for 15 doses
					CNS therapy: vincristine, doxorubicin, 6-MP, dexamethasone, PEG-asparaginase, IT MTX/ARA-C/hydrocortisone, cranial radiation
					Consolidation II, Cycle 1–8: doxorubicin, dexamethasone, vincristine, 6-MP, PEG-asparaginase, MTX
					IT MTX/ ARA-C/hydrocortisone
					Continuation therapy, Cycle 1–20: dexamethasone, vincristine, 6-MP, MTX

ALL: acute lymphocytic leukemia, AML: acute myeloid leukemia, APML: acute promyelocytic leukemia, MTX: methotrexate, ARA-C: cytarabine, HiDAC: high-dose cytarabine, 6-MP: 6-mercaptopurine, and ARTA: all-trans retinoic acid.

**Table 2:** Timing of chemotherapy, hormonal treatments, and menstrual change.

Subject	Age	Time since chemotherapy (years)	Time between menarche and chemotherapy (years)	Menstrual change during chemotherapy	Time to return of menses (months)	Duration of COC (months)	Duration of GnRH agonist (months)	Menstrual change after chemotherapy
1	39	3.7	23	Stopped	9–12	0	Unknown	Lighter
2	33	4.0	18	Stopped	<1	0.5	3	Restarted and returned to normal
3	33	2.4	18	Stopped	>12	0	31	Restarted and returned to normal
4	27	0.5	14	More frequent	<1	24	6	Lighter
5	25	1.8	12	Stopped	6–9	0	24	Lighter

COC: combined oral contraception. GnRH agonist: gonadotropin releasing hormone agonist.

**Table 3:** Ovarian reserve markers in the leukemia and comparison groups.

Group	Subject	Age (years)	Follicle stimulating hormone (mIU/mL)	Estradiol (pg/mL)	AMH (ng/mL)	Inhibin B (pg/mL)	Antral follicle count	Ovarian volume (cm^3^)
L	1	39	15.7	20.2	0.05	45.3	7	1.8
C	1	38	71.2	75.6	<0.02	29.3	6	7.3

L	2	33	6.2	96.5	1.87	18.9	13	4.7
C	2	34	5.4	32.2	3.5	68.6	28	11.8

L	3	33	15.6	24.6	<0.02	116	3	9.6
C	3	34	5.0	27.6	4.4	57.9	19	3.6

L	4	27	10.0	28.8	1.86	36.5	12	1.8
C	4	27	9.1	33.6	2.8	84.8	17	7.6

L	5	25	7.7	37.2	0.69	23.9	14	4.1
C	5	26	8.8	44.6	3.14	114	20	7.3

L	Median (Range)		10.0 (6.2–15.7)	28.8 (20.2–96.5)	0.69 (0.02–1.87)	36.5 (18.9–116)	12 (3–14)	4.1 (1.8–9.6)
C	Median (range)		8.8 (5.0–71.2)	33.6 (32.2–75.6)	3.5 (0.02–4.4)	68.6 (29.3–114)	19 (6–28)	7.3 (3.6–11.8)

L: leukemia group; C: comparison group. Matched subjects have the same number. Ovarian volume is the average volume of the right and left ovary.
